# A transition of care model from hospital to community for Hispanic/Latino adult patients with diabetes: design and rationale for a pilot study

**DOI:** 10.1186/s40814-022-01203-z

**Published:** 2022-12-05

**Authors:** Leonor Corsino, Blanca Iris Padilla

**Affiliations:** 1 Department of Medicine Division of Endocrinology, Metabolism and Nutrition, Department of Population Health Sciences, Duke School of Medicine, Durham, NC 27710 USA; 2 Duke University School of Nursing, Duke Regional Hospital, Diabetes Management Service, Durham, NC 27710 USA

**Keywords:** Hispanic/Latino, Diabetes, Transition, Hospitalization, Community

## Abstract

**Background:**

The Hispanic/Latino population is disproportionately affected and has a higher risk of developing diabetes than their non-Hispanic White counterparts and worse diabetes-related outcomes. Diabetes continues to be an economic burden. This economic burden is partially due to the significantly higher rates of hospital readmission for individuals with diabetes. People with diabetes, particularly those who are members of racial/ethnic minority groups, are at higher risk for readmission and emergency department (ED) visits. Despite recommendations regarding transition of care, an optimal approach to the transition of care for ethnic/minority patients remains unclear.

**Methods:**

The study population includes self-identified Hispanic/Latino adults with diabetes. We have two aims: (1) designed and developed a transition of care model and (2) pilot test the newly developed transition of care model. For aim 1, semi-structures interviews conducted with patients and providers. For aim 2, patients admitted to the hospital enrolled to receive the newly designed transition of care model. For aim 1, patients and providers completed a short questionnaire. For aim 2, patients completed a set of questionnaires including demographic information, medical history, sociocultural, and social support. The primary outcome for aim 2 is emergency department visit within 30 days post-discharge. The secondary outcome is 30- days unplanned readmissions. Feasibility outcomes include the number of participants identified, number of patients enrolled, number of participants who completed all the questionnaires, number of participants with a 30-day follow-up call, and number of participants who completed the 30-day post-discharge questionnaire. Due to the COVID-19 pandemic, the study design was adapted to include the Plan-Do-Study-Act framework to adjust to the ongoing changes in transition of care due to the pandemic burden on the health care systems.

**Conclusion:**

Transition of care for Hispanic/Latino patients with diabetes remains a major area of interest that requires further research. The pandemic required that we adapted the study to reflect the realities of health care systems during a time of crisis. The methods share in this manuscript can potentially help other investigators as they designed their studies.

**Trial registration:**

ClinicalTrials.gov identifier NCT04864639. 4/29/2021. https://clinicaltrials.gov/ct2/show/NCT04864639.

**Supplementary Information:**

The online version contains supplementary material available at 10.1186/s40814-022-01203-z.

## Key messages regarding feasibility

1. Uncertainties regarding feasibility
  Time and resource constraints due to pandemic
2. Key findings
  Level of support needed from the health system
3. What are the implications of the feasibility findings for the design of the main study?
  Marginalized patients (especially non-English speakers) continue to face significant challenges pertaining to transition of care from the hospital to the community.

## Background

Diabetes mellitus is a complex chronic condition that has reached epidemic proportions in the USA and globally. In 2020, an estimated 34.2 million people, approximately 10.5 % of the US population, had diagnosed diabetes [1]. Among racial/ethnic minority groups, the Hispanic/Latino population are disproportionately affected and have a higher risk of developing diabetes than their non-Hispanic White counterparts [2, 3] and worse diabetes-related outcomes [4]. Diabetes continues to be an economic burden in the USA; it is directly responsible for more than one-eighth of US health care expenditures. In 2017, the total cost of diabetes was $327 billion, representing a 26% increase over the past 5 years [5]. This increase in the economic burden is partially due to the significantly higher rates of hospital readmission for individuals with diabetes compared to individuals without diabetes [4–8].

According to the Center of Disease (CDC), in 2018, there were 8.25 million hospital discharges with diabetes as any listed diagnosis among adults aged 18 years or older [1]. People with diabetes, particularly those who are members of racial/ethnic minority groups or uninsured/underinsured, or who have a lower socioeconomic status, a history of prior admission, associated comorbidities, or an emergent or urgent admission, are at higher risk for readmission and emergency department (ED) visits than people without diabetes [6, 9]. Despite national recommendations regarding transition of care, an optimal approach to the transition of care for ethnic/minority patients remains unclear [4]. Diabetes is one of the most common conditions presented and managed in the acute care setting, yet there is a paucity of literature regarding best practices for transitioning patients with diabetes to the outpatient setting, especially those in the Hispanic/Latino population [6, 10].

Patients transitioning from the hospital to the community are in a vulnerable state, and this is particularly true of those with chronic conditions such as diabetes that require self-care management. The American Diabetes Association (ADA) recommends a well-structured, patient-centered transition of care discharge plan with a follow-up appointment within a month of discharge to reduce the rates of readmission and length of subsequent hospital stay. Unfortunately, achieving such a transition is significantly challenging for patients with fewer resources, cultural and language barriers, and low health literacy; these factors increase the risk for visits to the ED and readmission [6]. Few studies to date have been conducted to address this important aspect of diabetes care for Hispanic/Latino’s patients with diabetes. Further, the limited available data examining post-discharge outcomes in patients with diabetes demonstrates that being Hispanic/Latino is associated with a higher risk for multiple hospitalizations and rehospitalization [11]. Given the ADA’s recommendation and the current gap of culturally appropriate transition of care models designed for Hispanic/Latino adults with diabetes, this pilot feasibility study aims to develop a transition of care model for Hispanic/Latino adults with diabetes discharged from the hospital to the community. This manuscript describes the study rationale, aims, design, and methods of a 2-year pilot feasibility study focused on developing and testing a transition of care model from hospital to community for Hispanic/Latino adult patients with diabetes.

### The present study

This study is funded by grant P30 DK111022-05 from the National Institute of Health (NIH)/National Institute of Diabetes and Digestive and Kidney Diseases (NIDDK). The study is approved by the University Institutional Review Board (IRB). The study is registered on the United States National Institutes of Health Clinical Trials Registry (ClinicalTrials.gov identifier NCT04864639), available online at: https://clinicaltrials.gov/ct2/show/NCT04864639.

The PIs and all members of the research team have ensured and will continue to ensure that all data collected is kept confidential. Each participant is assigned a study number which is solely for administrative purposes. Data is stored in an encrypted, password-protected database. No participant data will be identifiable in any publication or report.

### Setting

The study was conducted in two private hospitals; the main one is an academic tertiary care facility and the second one is a general service hospital which is part of the larger health system. Each of the hospitals are in a county where 14% of the population is Hispanic/Latino.

## Methods and design

In year 1, a transition of care model was designed and developed. This was achieved by gathering qualitative data through semi-structured interviews with (a) Hispanic/Latino patients with diabetes who had had a recent (within the past 3 months) hospitalization, and (b) outpatient and inpatient health care providers. The information collected provided a greater understanding of the challenges and barriers experienced by Hispanic/Latino adult patients while transitioning from the hospital to the community. In year 2, the study will focus on pilot testing the new transition of care model. This will be accomplished by using a 2-arm approach and randomizing 32 adult Hispanic/Latino patients with diabetes who have been admitted to the hospital. One arm will enroll in the usual transition of care process, and the second arm will enroll using the newly developed transition of care model. During the period of the study design, the usual transition of care consisted of an electronic generated discharge summary which included, but not limited to the admission diagnosis, medication list (new and discontinued), a list of the follow-up visits, diagnostic testing(s), and laboratory results.

**Fig. 1:**
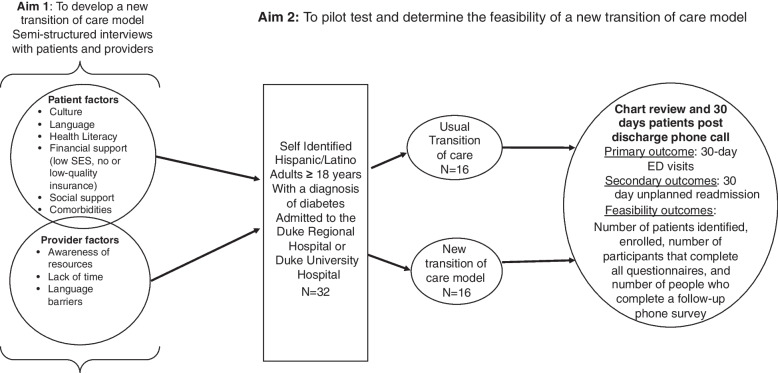
Overarching study conceptual framework and initial study design

Figure 1 provides details of the pilot study framework including structure, process, and outcomes. The 2019 coronavirus (COVID-19) pandemic has led to unprecedented challenges within our health system, academic institution, community, and broader society. Given mandated physical distancing and national lockdowns, data collection was modified from face-to-face to remote data collection for Aim 1, using Zoom as the method accepted by our institution.

### Study aims

Our first aim is to develop a model for transition of care from the hospital to the community for Hispanic/Latino adult (≥18 years of age) patients with diabetes, taking into consideration patient and provider perspectives. Our second aim is to pilot test and determine the feasibility of a transition of care model for Hispanic/Latino adult patients (≥18years of age) with diabetes.

### Participants and inclusion criteria

For aim 1, study participants included self- identified Hispanic/Latino adult patients with diabetes and health care providers. Inclusion criteria for patients are the following: (a) adult (age ≥18 years) and (b) self-identified Hispanic/Latino with a diagnosis of diabetes and a recent (within the past 3 months) hospitalization. There are no exclusion criteria based on health status, comorbidities, or other factors. Inclusion criteria for providers are the following: clinical and/or hospital provider (e.g., Doctor of Medicine [MD] or Osteopathic Medicine [DO], Nurse Practitioner [NP], Physician Assistant [PA]) currently practicing in the inpatient and/or outpatient setting. We aimed to enroll health care providers that represent the diversity of providers taking care of patients with diabetes in the inpatient and outpatient setting.

For aim 2, participant inclusion and exclusion criteria are identical to those for aim 1 but include an additional criterion: patients currently admitted to the hospital with anticipated discharge to the home setting.

Enrollment for aim 1 was initially intended to be accomplished by word of mouth and referral by local Hispanic/Latino organizations; however, to ensure that participants enrolled included those with a recent hospitalization, a purposeful sampling technique was used to identify potential participants using the health system’s electronic medical record (EMR). This method was selected as it allowed for the identification and selection of information-rich participants, and the most effective use of limited resources. Those meeting inclusion criteria were approached by telephone and screened to determine interest and obtain informed consent. Provider recruitment was initially to be accomplished by posting flyers at the target hospitals and clinics and by word of mouth; however, due to restrictions imposed by the COVID-19 pandemic, word of mouth and emails were used as the contact methods.

## Description of the study methods

This pilot and feasibility study was designed with two main goals:

1. Develop a new transition of care model based on data collected from participants (patients and providers) including but not limited to challenges and barriers to and facilitators of transition of care from the hospital to the community for adult Hispanic/Latino patients with diabetes.
2. Pilot test the newly developed transition of care model to determine its feasibility and the potential effect on ED visits and unplanned readmissions 30 days post-discharge.

### Procedures

Aim 1: Patients and providers participated in one-on-one semi-structured interviews scheduled at a time and date convenient for participants. Initially, the interviews were designed to be in-person and face-to-face; however, due to the COVID-19 pandemic, restrictions were imposed on in-person, noncritical research-related meetings. Given physical distancing measures, national lockdowns, and other restrictions, the traditional face-to-face qualitative interview method was not possible, and interviews were conducted remotely using Zoom or by telephone. The intended recruitment goal was 12 patients and 8 providers or until data saturation was reached.

The semi-structured interviews were designed to gather information pertaining to patients’ and providers’ perspectives of the current transition of care of patients with diabetes from the hospital to the community setting. Questions were developed using three types of questions: engagement, exploratory, and exit (see Additional file 1)*.* All participants provided written informed consent prior to their interview. Patients completed a short demographic questionnaire including the age, preferred language, place of birth, years in the USA (if applicable), marital status, gender, insurance, diabetes type, years since diagnosis of diabetes, comorbidities (i.e., hypertension, hyperlipidemia, coronary artery disease, obesity, other), recent hospitalization within the past 30 days and year, visit to the ED within the past year, data regarding recent hospitalization, date of recent hospitalization, duration of the most recent hospitalization, and education. Providers completed a short demographic questionnaire including age, preferred language, place of birth, years in the USA (if applicable), practice location, highest degree, years in practice, practice setting (inpatient vs outpatient), subspecialty, percentage of patients who are Hispanic/Latino, percentage of patients with diabetes, gender, ethnicity, and race.

All interviews were recorded and transcribed by the two study investigators.

Aim 2. The goal of this aim is to randomize 32 participants admitted to the hospital with diabetes into two arms: (1) the usual transition of care and (2) a transition of care model newly developed using information obtained in Aim 1. To determine our sample sizes, we used published data in the generalize diabetes population. Yan et al. reported that 18.7% of their study sample of patients with hyperglycemia had an emergency room re-visit within 30 days [12]. Since having hyperglycemia is the characteristic of diabetes and could be an indicator of distress for patients with diabetes, the estimate is a reasonable expectation for our study [13]. For this pilot study, we want to ensure that we can at minimum detect a difference in the primary outcomes (ED re-visits) between the two examined groups. Considering this is a pilot and feasibility study that will inform future larger-scale randomized trials, we estimated a total sample of 28 to 61 participants [14]. For our study’s goals and the resources available, we will use the lowest of the range, 28 (14 per group), as our study’s sample size. Based on this study design, very little attrition is expected. However, we will enroll 2 additional participants per group for a sample total of 32.

### Outcomes

Table 1 displays the measures for aim 2.Measures included in the study assessments are adapted from the NIH-sponsored Hispanic Community Health Study/Study of Latinos [15] and the My Bridge (Mi Puente) study [16].

**Table 1:** Measures and time points

Measure	Baseline	30 days post-discharge
**Aim 2**		
Personal information	X	
Medical history	X	
Sociocultural ^a^	X	
Single Item Measure of Social Support (SIMSS)^a^	X	
Single Item Literacy Screener (SILS)^a^	X	
HealthCare Access and Barriers ^a^	X	
30 days post-discharge phone call		X
Qualitative questions ^b^		X
30 days post-discharge chart review		X

*Abbreviations*: *DOB* date of birth, *PCP* Primary Care Provider

^a^Adapted from mi Puente trial

^b^5 participants will be enrolled for aim 2 to be re-interviewed (see new direction)

The feasibility outcomes include (1) the number of participants identified using the electronic medical records that meet inclusion criteria, (2) the number of patients approached and enrolled, (3) the number of participants who completed all baseline questionnaires, (4) the number of participants that completed the 30-day follow up call, and (5) the number of participants who answered the 30-day post-discharge questionnaire. Feasibility outcomes are tracked by the study team using web-based data management software and an internally developed tracking document.

*The number of participants identified:* using the institutional electronic medical record the study coordinator runs a weekly query using the inclusion criteria for the study. The study team reviews the medical records of potential participants emerging from the weekly query to confirm their qualifications. Patients that qualified based on the initial review and are admitted to the hospital are approached by the study team, in person, to further confirm they meet the inclusion criteria, to determine their interest in the study, and obtain informed consent.

*The number of patients approached and enrolled:* records are kept of each prospective participant approached by the study team including those that declined participation and those that enrolled after signing the informed consent.

Each participant enrolled in the study is assigned a record ID on the web-based data management site. The data management site facilitates data collection and tracking of record completion including tracking the number of participants who enrolled and completed informed consent, the number of participants that completed all baseline questionnaires, the number of participants that completed the 30-day follow-up call, and the number of participants who answered the 30-day post-discharge questionnaire. The web-based data management site provides a visual tool to facilitate tracking of each study procedures. Each completed task is indicated in the record in green. A dashboard allows investigators to run a statistical summary of completed tasks by each enrolled participant. We use this information to compare the number enrolled versus the number completing follow-up questionnaires and calls to determine retention in the study. To further determine the feasibility of the study, the investigators examine and review the number of participants identified in the initial query versus numbers approached versus numbers enrolled. Also, we explore the number enrolled per week to extrapolate the potential number of participants that can be enrolled per week and month. We also review time spent per study team reviewing charts, approaching participants, obtaining informed consent, and completing the questionnaires at baseline and 30 days.

Feasibility outcome data will provide essential and critical information about the recruitment and retention of the study target population in a larger trial. Lessons learned from this pilot and feasibility study will inform the development of a process for the recruitment, retention, data collection, testing, and implementation of the proposed new transition of care model in a statistically powered randomized clinical trial.

### New direction

The COVID-19 pandemic has created many challenges for the Hispanic/Latino community, which has suffered from the pandemic disproportionally compared to the overall percentage of residents in the area. As of March 2021, there were 12,353 documented cases of the virus per 100k Hispanic/Latino people in NC [17]. During the peak of the pandemic in 2020, Hispanic/Latino people accounted for approximately 65% of the COVID-19 cases in Durham, North Carolina, although only 14% of the county’s overall population is Hispanic/Latino [18].

Given the multiple challenges brought on by the COVID-19 pandemic, the PIs found it necessary to adapt and modify the original study design. Under the guidance of the study sponsors/mentors, the study design was modified in several ways. First, it included conducting a community consultation studio in collaboration with the university’s Clinical and Translational Science Institute/Community Engagement Research Initiative Core (CERI). This consultation provided additional data from the perspective of people living with and managing their diabetes while residing in the local community.

In addition to the consultation, the study design was changed from a randomized pilot study to a *Plan-Do-Study-Act (PDSA)* cycle framework. This model was chosen for several reasons: (a) the cycle can be used to test change over time and allows for adjustment during implementation to adapt to ongoing changes in health systems, (b) it facilitates implementation, and (c) it provides a more realistic assessment of the potential effect of a new transition of care to be implemented in the real word during challenging times [19].

The rationale for this modified approach was also informed by the fact that “the usual transition of care” was significantly altered to accommodate the changes implemented by hospitals during the pandemic due to increased numbers of patients admitted with COVID-19, limited beds and staff, and the overwhelming effect of the pandemic on health care systems in the country. These challenges rendered the “usual transition of care” no longer applicable, and it became apparent that we needed to adapt to the ongoing adjustments made by the health care system during the critical time of the pandemic.

**Fig. 2:**
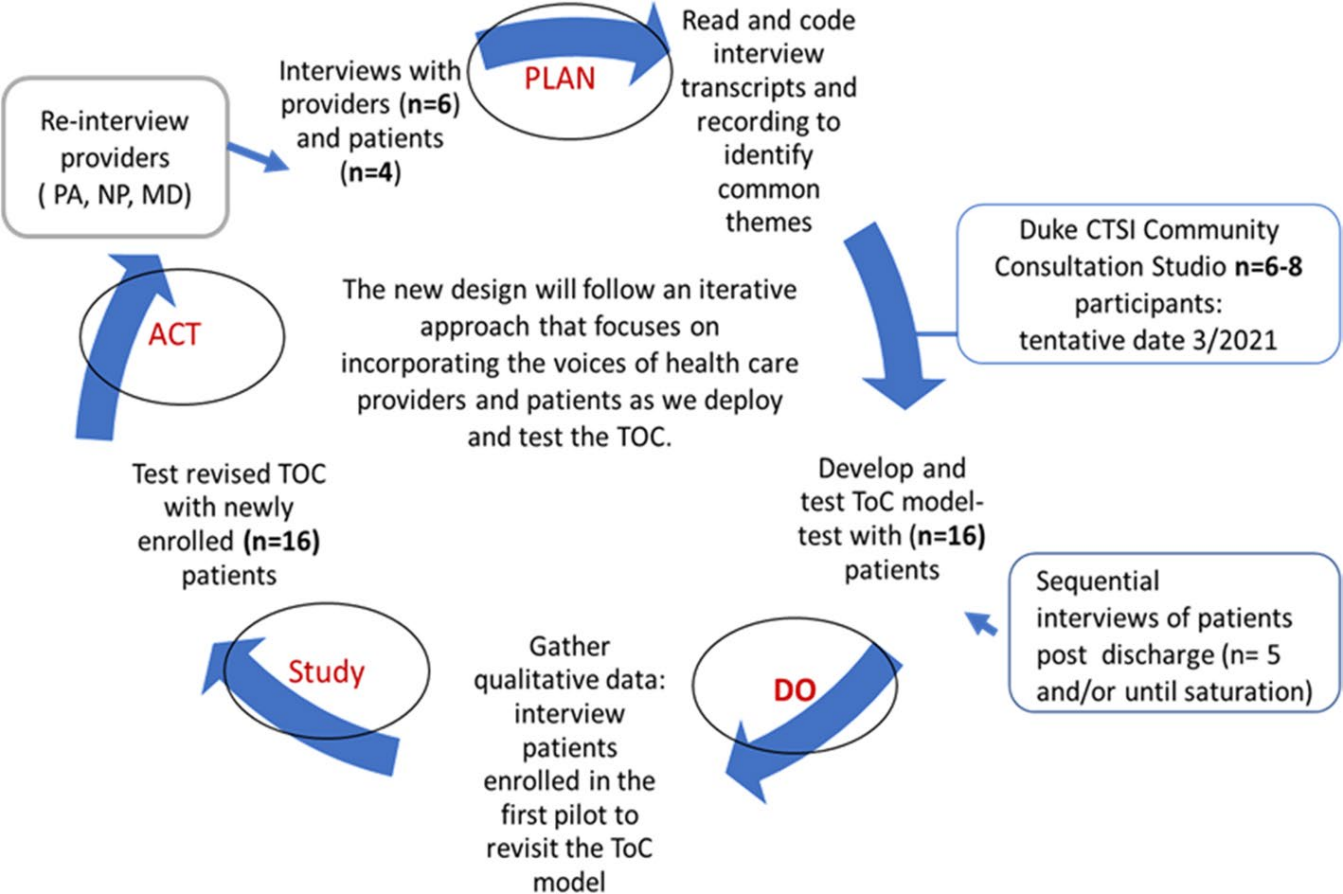
O Transition of Care (TOC) data collection using the PDSA framework

Using the PDSA framework, enrollment will include 16 unique patients admitted to the hospital, followed by semi-structured interviews of a total of 5 patients to revisit and revise the transition of care model. Subsequently, test the revised version of the model with an additional 16 patients. At the end of enrollment, we plan to interview a total of 3 providers to explore their perspectives of the new model. Figure 2 depicts the new study design using the PDSA framework.

### Statistical analysis

For aim 1, the PIs, both of whom are well experienced in qualitative research, transcribed the interviews into a Word document. The interviews were read multiple times for accuracy and emersion of the data and were coded into categories. The PIs reviewed the categories together and grouped the categories into common themes.

For aim 2, descriptive statistics will be used to summarize participants’ demographic characteristics. Means and standard deviations will be used for continuous variables, frequencies, and percentages for categorical variables overall and by enrollment group. A paired *t* test will be used to compare the two cohorts. The feasibility outcomes will be assessed to identify any differences between the groups and to refine the intervention.

## Discussion

This pilot study aims to address a critical gap in the transition of care literature, specifically regarding the high-risk population of Hispanic/Latino people with diabetes transitioning from the hospital to the community. During the discharge process, an abundance of information is provided to patients, especially to individuals with a new diagnosis of diabetes or who are new to an insulin regimen, as these patients require self-management of blood glucose, diet, and (potentially) insulin administration. The discharge process is further complicated when a language barrier is present, placing the patient at risk for adverse events. Providing effective delivery of discharge instructions is critical, especially for patients with chronic conditions such as a diabetes. Moreover, discharge instructions must be provided in a manner that is linguistically and culturally appropriate as well as easily understood by the patient and their family. A study noted the importance of optimizing communication for patients with limited English proficiency (LEP) at discharge, regardless of the patient’s primary language [20, 21]. Moreover, patients should receive written communication in their preferred language. It is important to consider that even when patients receive discharge instructions in their language of choice, a lack of health literacy can add a further layer of complexity to their ability to understand instructions.

The importance of providing simple instructions that are easy to understand may be evidenced by the fact that patients with diabetes from marginalized communities tend to have higher rates of readmission and ED visits, and higher costs for diabetes care [6]. These high rates of readmission and 30-day ED visits are potentially preventable. Research shows the benefits of providing clear recommendations pertaining to medication usage, diabetes education while the patient is admitted, and scheduled follow-ups 1–2 weeks post-discharge [22]. Despite the best efforts and the use of EMRs, marginalized patients (especially non-English speakers) continue to face significant challenges pertaining to transition of care from the hospital to the community. Finding ways to make such transitions more culturally acceptable could help to improve patients’ outcomes post-discharge. Evidence shows that culturally adapted interventions that address the heterogeneity within the Hispanic/Latino population are valuable for changing outcomes [23].

The success of an intervention such as the one being tested in this pilot study can inform larger clinical trials and process changes in real life which can be implemented to improve the care of Hispanic/Latino patients with diabetes.

This study has several strengths. First, the transition of care model is patient- and provider-driven. Although it will be developed based on current gaps in the literature, the participants’ (both patients with diabetes and providers) recommendations and qualitative feedback were used as the basis of the model. This will likely increase the success of the model while simultaneously increasing favorable patient outcomes. Secondly, testing the new model in a real-world setting can provide essential information to the PIs on how well the model performs. Finally, the model can adapt to the current COVID-19 pandemic and ongoing changes within the hospital setting such as the constant reassessment of the discharge process to ameliorate the current burden on health care systems.

### Study status

At the time of this manuscript’s submission, the research team is recruiting participants for aim 2.

## Supplementary Information

**Additional file 1.** Semi-structured interviews: patient and provider questions.

## Data Availability

Not applicable.
