# Supplementary material for: A transition of care model from hospital to community for Hispanic/Latino adult patients with diabetes: design and rationale for a pilot study
Source: Pilot Feasibility Stud. 2022 Dec 5;8:246. doi: 10.1186/s40814-022-01203-z (PMC9721061; doi:10.1186/s40814-022-01203-z)
Supplement: Supplementary file 1 — Additional file 1. Semi-structured interviews: patient and provider questions. [file 40814_2022_1203_MOESM1_ESM.docx]

**Additional file 1**

**Semi-structured Interviews: Patient and Provider Questions**

| **Patient Interview Questionnaire** | **Provider Interview Questionnaire** |
| --- | --- |
| **Engagement questions** (Health System): Think about the last time you were admitted to the hospital.   1. How long ago were you admitted to the hospital? 2. How long was your hospital stay? 3. In the last year, how do you think your diabetes has gotten in the way of your health related to your admissions? 4. Can you please share with us what was your experience about the discharge during your last hospital admission? 5. During your hospital stay, how do you think having diabetes contributed to your admission? 6. Can you share your experience regarding any barriers you may have had during your last discharge from the hospital? 7. What was your experience like when the nurse or other health care provider discharged you from the hospital? 8. Can you share any barriers to understanding the information given? | **Engagement questions** (Health System):   1. Can you describe the current discharge process specific to Hispanic/Latino patients with diabetes? 2. Based on your experience with [blinded] and the community setting, can you describe or give examples on some of the challenges you face, as a health care provider, when discharging Hispanic/Latino patients with diabetes? 3. What ideas do you have that may improve the current transition of care process for Hispanic/Latino patients with diabetes? |
| **Exploratory questions** (Social Contributors to Health) (Community and individual)   1. Thinking back to your last hospital discharge, can you remember what were your biggest challenges at that time? 2. Probes:    1. Why do you think those challenges occurred?    2. What do you think could have made a difference?    3. What were your thoughts and feeling during the process?    4. What stood out the most for you and why?    5. What fears or concerns did you have? 3. During the discharge, can you describe the type of information that was included? 4. Remembering back at the time of your last hospitalization, can you tell what it was like receiving education about diabetes, diabetes medicine, and your follow up care? 5. Can you describe the approach during the time you received the information? 6. What type of information do you think should have been included? Why? 7. What kind of information was provided to you regarding medicines, community resources, changes in your diet and exercise? 8. Were you given specific teaching about high and low blood sugar? 9. If yes,    1. Describe what was the most helpful information you received.    2. What parts or components of the information do you feel were appropriate for you?    3. For this question, can you describe how the following discharge instructions were given to you:       1. follow up visit with a health care provider       2. medication use and refill       3. diabetes-related information (e.g., diet, exercise, how often to check your blood sugar, how to take the medications, what to do if your blood sugar is high or low) 10. Describe how your friends and family were helpful during the discharge process. 11. Sometimes Hispanic/Latino patients face barriers when it comes to health care. Describe the barriers you faced, if any, and how you were able to work through them. 12. Do you have ideas on how those barriers could be eliminated? 13. Can you share some of the information that was provided to you on community resources to help you as a Hispanic/Latino with diabetes? | **Exploratory questions** (Community and Individual):   1. Based on your experience, what are some of the existing gaps that need to be addressed regarding the transitioning period for Hispanic/Latino patients when discharging from the hospital to the community? 2. What are some providers’ needs during the transitioning of care of Hispanic/Latino patients with diabetes from the hospital to the community? |
| **Exit question**  Can you please share any additional information or ideas on how the discharge process can be made easier? | **Exit question**  Can you please share any additional ideas you may have on how the transition from the hospital to home can be made more seamless for this population (Hispanic/Latino patients with diabetes)? |
